# Spiny dogfish, *Squalus suckleyi*, shows a good tolerance for hypoxia but need long recovery times

**DOI:** 10.1093/conphys/coae054

**Published:** 2024-08-13

**Authors:** Gudrun De Boeck, Isabelle Lardon, Marleen Eyckmans, Trung Nghia Vu, Kris Laukens, Roger Dommisse, Chris M Wood

**Affiliations:** ECOSPHERE, Department of Biology, University of Antwerp, Groenenborgerlaaan 171, 2020 Antwerp, Belgium; Bamfield Marine Sciences Centre, 100 Pachena Rd, Bamfield BC V0R 1B0, Canada; ECOSPHERE, Department of Biology, University of Antwerp, Groenenborgerlaaan 171, 2020 Antwerp, Belgium; Bamfield Marine Sciences Centre, 100 Pachena Rd, Bamfield BC V0R 1B0, Canada; INVE Aquaculture, Hoogveld 93, 9200 Dendermonde, Belgium; ECOSPHERE, Department of Biology, University of Antwerp, Groenenborgerlaaan 171, 2020 Antwerp, Belgium; Bamfield Marine Sciences Centre, 100 Pachena Rd, Bamfield BC V0R 1B0, Canada; Pharmaceutical, Biomedical and Veterinary Sciences, Universiteitsplein 1, 2610 Wilrijk, Belgium; Adrem Data Lab, Department of Computer Science, University of Antwerp, Middelheimlaan 1, 2020 Antwerp, Belgium; Department of Medical Epidemiology and Biostatistics, Karolinska Institutet, Nobels väg 12A, 171 65 Solna, Sweden; Adrem Data Lab, Department of Computer Science, University of Antwerp, Middelheimlaan 1, 2020 Antwerp, Belgium; Department of Chemistry, University of Antwerp, Groenenborgerlaan 171, 2020 Antwerpen, Belgium; Bamfield Marine Sciences Centre, 100 Pachena Rd, Bamfield BC V0R 1B0, Canada; Department of Zoology, University of British Columbia, 6270 University Blvd, Vancouver, BC, V6T 1Z4, Canada; Department of Biology, McMaster University, 1280 Main St. West, Hamilton, ON, L8S 4K1, Canada

**Keywords:** ^1^H-NMR spectroscopy, amino acids, critical oxygen pressure, elasmobranch, hypoxia, ketones, metabolomics, recovery, shark, Abbreviations: PCA principal component analysis, TMAO trimethylamine oxide

## Abstract

Pacific spiny dogfish, *Squalus suckleyi*, move to shallow coastal waters during critical reproductive life stages and are thus at risk of encountering hypoxic events which occur more frequently in these areas. For effective conservation management, we need to fully understand the consequences of hypoxia on marine key species such as elasmobranchs. Because of their benthic life style, we hypothesized that *S. suckleyi* are hypoxia tolerant and able to efficiently regulate oxygen consumption, and that anaerobic metabolism is supported by a broad range of metabolites including ketones, fatty acids and amino acids. Therefore, we studied oxygen consumption rates, ventilation frequency and amplitude, blood gasses, acid–base regulation, and changes in plasma and tissue metabolites during progressive hypoxia. Our results show that critical oxygen levels (*P*_crit_) where oxyregulation is lost were indeed low (18.1% air saturation or 28.5 Torr at 13°C). However, many dogfish behaved as oxyconformers rather than oxyregulators. Arterial blood PO_2_ levels mostly decreased linearly with decreasing environmental PO_2_. Blood gases and acid–base status were dependent on open versus closed respirometry but in both set-ups ventilation frequency increased. Hypoxia below P_crit_ resulted in an up-regulation of anaerobic glycolysis, as evidenced by increased lactate levels in all tissues except brain. Elasmobranchs typically rely on ketone bodies as oxidative substrates, and decreased concentrations of acetoacetate and β-hydroxybutyrate were observed in white muscle of hypoxic and/or recovering fish. Furthermore, reductions in isoleucine, glutamate, glutamine and other amino acids were observed. After 6 hours of normoxic recovery, changes persisted and only lactate returned to normal in most tissues. This emphasizes the importance of using suitable bioindicators adjusted to preferred metabolic pathways of the target species in conservation physiology. We conclude that Pacific spiny dogfish can tolerate severe transient hypoxic events, but recovery is slow and negative impacts can be expected when hypoxia persists.

## Teaser

Pacific spiny dogfish tolerate low oxygen levels relatively well but show large individual variation in coping strategy (oxyregulators versus oxyconformers). Lactate, a classic indicator of anaerobic metabolism, reflected the onset of hypoxia but was not a good biomarker of the recovery time needed.

## Introduction

Environmental hypoxia occurs in coastal and open ocean marine ecosystems and varies both spatially and temporally, affecting food chains and survival and distribution of marine fish species worldwide (Rabalais *et al*., 2010; Carstensen *et al*., 2014; Gobler and Baumann, 2016; Breitburg *et al*., 2018; Waller *et al*., 2024). Hypoxic zones are expanding due to climate change combined with increasing loads of nutrients from agriculture, sewage and industrial waste. This concerning trend is recognized in the reports of the Intergovernmental Panel on Climate Change (Bindoff *et al*., 2019; IPCC, 2019) and was at the origin of the IOC-UNESCO Global Ocean Oxygen Network (GO_2_NE) and the Global Ocean Oxygen Decade (GOOD) program of the UN Ocean Decade (2021–2030). A report of the Global Ocean Oxygen Network shows that the area of low oxygen water in the open ocean has increased by 4.5 million km^2^ since the 1960s, and over 500 low oxygen sites have been identified in estuaries and other coastal water bodies (Intergovernmental Oceanographic Commission, 2018).

Hypoxic areas are commonly observed in shallow and open ocean areas and thus fish may regularly encounter hypoxic stress threatening growth and survival. To date, a number of studies investigated responses to oxygen limitation in elasmobranch fish. These range from the classical work by Piiper *et al*. (1970) and Butler and co-workers (Butler and Taylor, 1975; Metcalfe and Butler, 1984) in the seventies and eighties using the greater and lesser spotted dogfish *Scyliorhinus stellaris* and *Scyliorhinus canicula* (belonging to the family of the cat sharks) up to the recent surge of studies on the anoxia tolerant epaulette shark (*Hemiscyllium ocellatum*) (reviewed in Milsom and Taylor, 2016). The latter coral reef dwelling shark is of particular interest due to its extreme tolerance of hypoxia and anoxia at tropical temperatures (close to 30°C). Some other species that often encounter hypoxia live in the oxygen minimum zone such as the migrating bigeye thresher shark, *Alopias superciliosus*, or deep-sea sharks such as the lollipop catshark, *Cephalurus cephalus*, which evolved such large gill surface areas to enhance oxygen uptake that their heads have a widened ‘helmeted’ or ‘lollipop’ appearance (Compagno, 1990; Wootton *et al*., 2015). However, in general our knowledge is limited, and a better understanding of hypoxia tolerance can contribute to better conservation of shark species that are threatened worldwide (Waller *et al*., 2024). Indeed, the last half century has seen an 18-fold increase in relative fishing pressure which has resulted in a 71% decline in global abundance of oceanic sharks and rays (Pacoureau *et al*., 2021) and a widespread depletion of reef sharks leading to their absence on 19% of the world’s tropical coral reefs (MacNeil *et al*., 2020). In order to effectively guide and improve conservation efforts, knowledge on the behaviour, ecology and physiology of sharks and their relatives is urgently needed (Waller *et al*., 2024). Despite their pivotal role in the marine ecosystem (Dulvy *et al*., 2021; Ferretti *et al*., 2010; Roff *et al*., 2016) and their circumglobal distribution, many questions remain unresolved, largely due to inherent logistic issues (e.g. large size, K-selected life history, migratory behaviour).

One of the parameters that has been used to assess hypoxia tolerance in fishes is the critical oxygen tension *P*_crit_. In progressive hypoxia fish are assumed to be oxyregulators down to *P*_crit_, meaning that they manage to maintain their oxygen consumption rates stable. Below *P*_crit_ they become oxyconformers and oxygen consumption rates drop with decreasing environmental oxygen (Rogers *et al*., 2016). A low *P*_crit_ is often thought to be associated with greater hypoxia tolerance as the fish can sustain its metabolic rate at low oxygen concentrations, and this is thought to reflect the binding affinity or P_50_ of the fish’s haemoglobin (Dejours, 1981; Speers-Roesch *et al*., 2012a; Morrison *et al*., 2016). Except for the epaulette shark with a P_crit_ of 2.2 mg O_2_ L^−1^ or 50 Torr at 25°C (Routley *et al*., 2002), most sharks have a high *P*_crit_ as seen in the bamboo shark (*Hemiscyllium plagiosum*; Chan and Wong, 1977), catsharks (Piiper *et al*., 1970; Butler and Taylor, 1975), blacktip reef shark (*Carcharhinus melanopterus*) and the shovelnose ray (*Rhinobatus typus*) (Routley *et al*., 2002). However, besides the catsharks, most of these studies are performed on tropical species. In the more temperate catsharks, oxyconforming or oxyregulation was seen to be temperature dependent. At colder temperatures (8°C), embryos of the lesser spotted dogfish *S. canicula* were oxyconformers while at 12–18°C they switched to oxyregulation (Thomason *et al*., 1996). Also adults of the same species oxyregulated at 17°C, but became oxyconformers between 12 and 17°C (Butler and Taylor, 1975). It is presently unknown whether this capacity to switch between oxyconforming and oxyregulation as observed in the catsharks is shared with other temperate species.

Pacific spiny dogfish (*Squalus suckleyi*) also live in temperate waters and have a preferred temperature range of 7–15°C (Ebert, 2003). Previous studies showed that severe hypoxia led to reduced urea retention and impaired acid–base regulation and ionoregulation, likely reflecting increased functional gill surface area (Zimmer and Wood, 2014). Not only does functional gill area increase in spiny dogfish, but reduced oxygen levels additionally affected ventilation rates. Increases in ventilation amplitude and frequency were seen in Pacific spiny dogfish (Perry and Gilmour, 1996; Acharya-Patel *et al*., 2018), and increased ventilation frequencies were also seen in lesser spotted dogfish (Metcalfe and Butler, 1984) and epaulette shark (Routley *et al*., 2002) during hypoxia. These responses are instrumental in oxyregulation, or maintaining a stable oxygen uptake down to the P_crit_, and therefore suggest that the Pacific spiny dogfish is an oxyregulator even at the low temperatures of 10–12°C where these studies were performed.

The use of the P_crit_ as a reliable index of hypoxia tolerance is controversial (Wood, 2018; Regan *et al*., 2019; Farrell *et al*., 2021) and other parameters have been suggested to give a better reflection of hypoxia tolerance (Mueller and Seymour, 2011; Speers-Roesch *et al*., 2013; Wood, 2018). Loss of equilibrium has been proposed as a relevant measure for hypoxia tolerance (Wood, 2018), but is not so easy to measure in benthic sharks as they tend to lie quietly on the bottom of respirometers. Lower lactate accumulation due to metabolic depression is also suggested to give a better prediction of hypoxia tolerance and the capacity for metabolic depression than P_crit_ (Speers-Roesch *et al*., 2012b). Other metabolic processes might also undergo changes during hypoxia, especially when it progresses slowly allowing the fish to physiologically adjust. Indeed, elasmobranchs have an exceptional metabolism compared to teleost or bony fish, in that they do not rely on the use of fatty acid oxidation but rather on lipid-derived ketone bodies (Ballantyne, 1997). The nitrogen quotient of elasmobranchs is generally high, which indicates a reliance on amino acids as an energy source (Wood *et al*., 2007; Speers-Roesch and Treberg, 2010; Giacomin *et al*., 2017). Plasma glutamine levels in elasmobranchs are among the lowest in fish due to the need for glutamine for urea synthesis but also for oxidation by mitochondria in heart and red muscle (Chamberlin and Ballantyne, 1992). How elasmobranchs metabolism responds under hypoxic conditions is presently a knowledge gap.

The aim of our study was twofold. First, we tested whether the Pacific spiny dogfish (*S. suckleyi*) is indeed an oxyregulator, even at cold temperatures, as is suggested by previous studies (Perry and Gilmour, 1996; Zimmer and Wood, 2014; Acharya-Patel *et al*., 2018). Therefore, we examined oxygen consumption rates (MO_2_) at decreasing environmental oxygen levels. Additionally, we hypothesized that the onset of anaerobic metabolism in spiny dogfish is linked to internal hypoxia. We predicted that changes in blood gasses and acid base status (arterial O_2_, CO_2_, pH) as well as the appearance of metabolites of anaerobic metabolism such as lactate will precede changes in ventilation and the P_crit_. Therefore, we measured ventilation frequency and amplitude, blood gasses, acid–base regulation and plasma lactate during slowly progressing hypoxia using open and closed respirometry systems. CO_2_ builds up in the latter, but not in the former, therefore we predict that effects on ventilation would show up earlier in the closed set-up as arterial PCO_2_ and associated pH decreases are also known to induce hyperventilation (Perry and McKendry, 2001; Rogers *et al*., 2016). In the second part of this study, we investigated the tissue metabolic responses under a continuous (2 and 6 h) hypoxia exposure and subsequent normoxic recovery (6 h). The chosen level of hypoxia was 60% of the estimated P_crit_. Due to the unique metabolism of elasmobranchs, plasma lactate might not be the most suitable indicator of the onset of anaerobic metabolism and recovery thereafter. We predicted that changes under hypoxia would not only occur in glucose and lactate levels, but also in ketones, fatty acids and amino acids. Therefore, qualitative high-resolution ^1^H-NMR spectra were obtained from polar extracts of gill, rectal gland, liver, white muscle and brain tissue to identify significantly altered metabolites. Since this was a profound, relatively long-lasting exposure, we hypothesized that changes in metabolites would be visible in all tissues and that recovery times might be compound-specific and tissue specific.

Consequently, the results of this study offer a comprehensive picture of the hypoxia tolerance mechanisms in Pacific spiny dogfish, *S. suckleyi*.

## Materials and methods

Experiments were conducted at the Bamfield Marine Sciences Centre, British Columbia, Canada. Male Pacific spiny dogfish, *S. suckleyi* were caught by angling in the proximity of the Bamfield inlet (48.83557 N, 125.13557 W) under Fisheries and Oceans Canada collecting permit XR2392015). They were subsequently kept in a large concrete indoor tank (151 000 l) continuously supplied with running aerated Bamfield Marine Station seawater (12–13°C, salinity 30‰) and were able to acclimatize for at least two weeks before the start of the experiments. Fish were fed twice a week with commercially purchased frozen hake (*Merluccius productus*) but fasted for at least 3 days prior to and for the duration of the experimental period. All experiments were carried out under the guidelines of the Canada Council for Animal Care under approval of animal care committees at BMSC and the University of British Columbia (joint AUP A14–0251).

Three series of experiments were conducted. In a first respirometry series, dogfish were submitted to closed respirometry. Respiration rates were followed as oxygen levels dropped to hypoxic levels to determine the critical oxygen concentration (P_crit_). Subsequently, the same procedure was followed using cannulated dogfish, and ventilation rates and blood gas levels were followed. In a second series of experiments, the closed respirometry set-up was replaced by an open set-up and N_2_ bubbling was used to reduce the oxygen levels. Ventilation rates and blood gas levels were determined as hypoxia increased at a comparable rate as measured during closed respirometry. Lastly, dogfish were subjected to prolonged hypoxia (2 and 6 h) and tissue samples were taken before and during hypoxia and after a 6-h recovery period for a metabolomics study.

### First experimental series: respirometry, ventilation and blood gasses in a closed set-up

#### Respirometry

In total 10 adult dogfish with a weight of 1.85 ± 0.10 kg were caught one-by-one from the holding tank and placed into individual polyurethane-coated wooden (105 × 18 × 30 cm, containing 54 L) or metal (120 × 30 × 60 cm, containing 75 L) boxes, filled with well aerated Bamfield Marine Station seawater (13°C, 30‰) with a flow-through of 1 L min^−1^ and left to habituate overnight. The next morning, the water surface was covered with a Styrofoam lid which tightly fitted in the box, aeration was stopped and an oxygen probe (CellOx 325 probe, WTW Oxi 3400i meter, Weilheim, Germany) was inserted through the lid. Oxygen consumption rates (*Ṁ*O_2_) were followed over approximately the next 3 hours until deep hypoxia (<10% saturation or < 16 Torr) was reached after which measurements were stopped, water was reoxygenated and fish were allowed to recover. Values of ṀO_2_ were calculated per body mass (kg) and over time (h).

#### Ventilation and blood gases

In total, five adult dogfish with an average weight of 1.91 ± 0.13 kg were caught one-by-one from the tank, and immediately anaesthetised in a 100 mg L^−1^ MS-222 (Sigma Chemicals, St. Louis, MO, USA) seawater solution neutralized with NaHCO_3_ (pH 7.8) for surgery. Methods followed those of De Boeck and Wood (2015). Dogfish were placed on a V-shaped operating table, and their gills were constantly irrigated with anesthetic throughout surgery. A small incision was made, approximately 5 cm anterior to the caudal fin, to the vertebrae, exposing the cartilaginous haemal canal. This canal was punctured with a #22 needle creating a small hole for a PE50 polyethylene cannula, filled with heparinised 50 i.u. ml^−1^ dogfish saline (6 mM NaHCO_3_, 257 mM NaCl, 7 mM NaSO_4_, 0.1 mM NaH_2_PO_4_, 4.1 mM KCl, 3 mM MgSO_4_, 5 mM glucose, 2 mM CaCl_2_, 350 mM urea, 15 mM TMAO). The cannula was inserted in the artery, which was verified by the presence of sufficient blood pressure to create a spontaneous blood flow even when the catheter was raised above the heart, and held in place by a sleeve of PE160 glued to the PE50 with cyanoacrylate tissue cement (3 M Vetbond, 3 M Animal Care Products, St Paul, MN, USA) and secured with two sutures to the skin. Additionally, a flared PE160 cannula was inserted in the branchial chamber between the first and second gill slit. It was held in place by a second short piece of flared PE240 which was glued to the PE160 cannula on the outside of the fish. The PE160 cannula was positioned in a 90° angle on the skin to allow the dogfish to breathe freely and was returned with a large loop towards the back of the dogfish where it was held in place by two sutures to the skin just before the dorsal fin. Dogfish were revived by artificial ventilation with anaesthetic-free seawater and were left to recover overnight in covered black Plexiglas fish boxes. The boxes were 98 cm in length, 20 cm in width and 25 cm in height and contained 47.5 l Bamfield Marine Station seawater (13°C, 30‰) with a flow-through of 0.75 L min^−1^ and were served with perimeter aeration.

The next morning, the water surface was covered, aeration was shut down and ventilation was measured at regular intervals as described below under analytical procedure as oxygen levels dropped. Immediately after a 2-min period of ventilation measurements a 0.6-ml blood was sampled using a 1-ml gas-tight Hamilton syringe, replaced with 0.6 ml saline and analysed as described below.

### Second experimental series: ventilation and blood gasses in an open set-up

Another five adult dogfish with an average weight of 1.91 ± 0.13 kg were caught one-by-one from the tank, and treated as in series 1 (see Ventilation and blood gases Section). In these five dogfish, the water surface was not sealed after overnight recovery but water was softly bubbled with N_2_ so that oxygen levels dropped approximately at the same rate as in the first series. Ventilation measurements and blood sampling were performed in an identical way.

### Third experimental series: hypoxia and recovery experiments

In addition, four experimental groups of spiny dogfish (1.84 ± 0.09 kg), a control group (*n* = 6), a 2-h (*n* = 5) and a 6-h hypoxic group (*n* = 5), and a group that was allowed to recover under normoxia for 6 h from the 6-h hypoxia exposure (recovery group, *n* = 5) were placed individually in metal boxes (volume of 75 L) to habituate overnight in well-aerated Bamfield Marine Station seawater at 13–14°C. The control animals were maintained at normal seawater oxygenation (100% air saturation, 158 Torr, 21 kPa or 8.8 mg O_2_ L^−1^). Hypoxia was induced gradually by bubbling nitrogen gas through the water to displace dissolved oxygen and by sealing the test box with rubber mats to avoid oxygen diffusion from air. Experimental animals were exposed to hypoxic conditions of 1 mg O_2_ L^−1^ (11–12% saturation or 18 Torr) for either 2 h or 6 h. The recovery group comprised fish that were exposed to 6 h of hypoxia and allowed to recover for 6 h in normoxic seawater. The dissolved O_2_ levels were monitored every 15 min using a WTW oxygen electrode (WTW oxi 3400i, Weilheim, Germany) and *M*O_2_ calculated per 15-min interval. Regulatory index (RI) was calculated according to Mueller and Seymour (2011). The RI provides a relative measure of regulatory ability. It is calculated by dividing the area under the *Ṁ*O_2_ versus PO_2_ curve above the linear trend (the area above the diagonal line of oxyconformation) by the total available area calculated in the same way for perfect oxyregulation (the same area up to the horizontal line of perfect regulation). This fractional index can vary from 0.0 (perfect oxyconformation, no area above the linear trend) to 1.0 (perfect oxyregulation).

At the completion of the experiments, dogfish were euthanized in an overdose of MS222 neutralised with NaHCO_3_ (1 g L^−1^, pH 7.8, Sigma Chemicals, St. Louis, MO, USA). Brain, liver, gills, white muscle and rectal gland tissue samples were subsequently excised rapidly on ice in this order, immediately snap-frozen in liquid nitrogen and stored at −80°C until further analysis at the University of Antwerp (at the Departments of Chemistry and Biology), Belgium.

### Analytical procedures

#### Ventilation measurements

For ventilation measurements, the branchial cannula was filled with sea water and connected to a pressure recording system which consisted of a pressure transducer (Statham P23BB, Statham Instruments, Oxnard, CA, USA), a transducer amplifier (Harvard Apparatus, Holliston, MA, USA), and an oscillograph (Harvard Apparatus, Holliston, MA, USA). The pressure transducer was calibrated directly by connection to a column containing different heights of water. This system allowed recording of ventilation frequency (breaths min^−1^) and the ventilation pressure amplitude (mmHg), as an index of stroke volume. Ventilation frequency was calculated as the frequency of breaths in one minute at the designated time. Ventilation amplitude was calculated as the average value of ten measurements of amplitude (randomly selected from periods of normal breathing, not using episodes of coughing or disturbance) at the designated time. Ventilatory index was calculated as frequency × amplitude (Giacomin *et al*., 2022).

#### Blood sample analysis

Blood samples of fish fitted with arterial cannulae were immediately analyzed for arterial pH_a_ and arterial oxygen tension (P_a_o_2_). The whole blood pH_a_ and P_a_o_2_ were measured at 13°C using a Radiometer GK2401C glass combination electrode coupled to a PHM82 standard pH meter (Radiometer Ltd, Copenhagen, Denmark), and a polarographic oxygen electrode coupled to an amplifier (Model 1900, A-M System, Everett, WA, USA), respectively. The electrodes were kept at the experimental temperature in chambers perfused with Bamfield Marine Station seawater. The remainder of the blood sample was immediately centrifuged (5 min at 10000 G), and plasma sub-samples were taken and kept at 5°C for determination of the levels of total CO_2_ concentration later that day. Plasma total CO_2_ was measured in duplicate on 50-μl samples using a Corning model 965 CO_2_ analyzer (Lowell, MA, USA). P_a_CO_2_ was calculated using the solubility of carbon dioxide (α_CO2_) and the apparent p*K* (p*K*_app_) for dogfish plasma according to Boutilier *et al.* (1984): P_a_CO_2_ = Cco_2_/(α_CO2_ (10^pH-pKapp^ + 1)) with Cco_2_ being total plasma CO_2_ concentration. Plasma [HCO_3_^−^]_a_ concentration was calculated as the difference between total plasma CO_2_ and α_CO2_ × P_a_CO_2_. In the series where hypoxia was induced by N_2_ bubbling, plasma lactate levels were determined using an enzymatic kit (R-Biopharm AG, Darmstadt, Germany).

#### Tissue extraction and metabolomics

Tissue extraction was carried out using the methanol/chloroform/water method as in Lardon *et al*. (2012, 2013) based on the protocols described by Lin and Wu (Lin *et al*., 2007; Wu *et al*., 2008). The aqueous tissue extracts were analysed on a Bruker Avance II-700 NMR spectrometer, operating at 700.13 MHz (Bruker Biospin, Europe), equipped with a 5-mm inverse TXI-Z probe and a BACS-60 automatic sample changer. Tuning, matching and shimming were performed automatically for each sample in order to minimize the variation due to sample manipulation.

One-dimensional ^1^H-NMR spectra of brain, liver, gills, white muscle and rectal gland extracts were measured at 25°C with a standard sequence using a 90° pulse (pulse sequence *zgpr*), a relaxation delay of 1.0 s with water presaturation, 64 scans collected into 16 k data points, a spectral width of 14 kHz and an acquisition time of 2.33 min per sample. Prior to Fourier transformation, all datasets were zero-filled to 32 k points and exponential line broadenings of 0.3 Hz were applied as well. Finally, all spectra were phase and baseline corrected and chemical shifts were referenced to sodium-3-trimethylsilylpropionate (TMSP) as an internal standard using the Topspin software (version 2.1, Bruker Biospin).

The identity of significant metabolites was assigned by comparison to tabulated chemical shifts in the literature (Fan, 1996) and confirmed by comparison to in-house and public databases (e.g. The Human Metabolome Database—HMDB, Chenomx).

The 1D spectra were converted to an appropriate format for subsequent statistical analysis by automatically segmenting each NMR spectrum in 0.05 ppm integrated spectral regions (bins or buckets) between 0.5 and 10 ppm using AMIX (Analysis of Mixtures, version 3.8.5, Bruker Biospin). Buckets from 4.70 to 5.0 ppm, containing the residual water resonance, were excluded. All spectra were mean-centred and were normalized to total intensity in order to reduce the influence of concentration variability among the samples.

### Statistical analyses

#### Respiration, ventilation and blood parameters

All data have been presented as mean values ± standard error (S.E.M.). The data were analyzed using GraphPad Prism 7.00. Two-tailed Pearson correlation tests were performed following linear regression. Regressions are shown with their 95% confidence intervals. For non-linear curves, segmented linear regressions with a least square fit were performed. A significance level of a *P*-value < 0.05 was used throughout.

#### Metabolomics analysis

The pre-processed NMR spectra were first subjected to principal component analysis (PCA) using AMIX, to reduce the dimensionality of the data and to obtain an overview by showing trends, clusters and potential outliers within the data sets. Data points were identified as outliers when they were not situated within the 95% confidence ellipse (Hotelling T^2^ multivariate profiling) (Lindon *et al.*, 2007) and outliers were subsequently removed from the data sets.

Following PCA and outlier detection and elimination, the created bucket tables of the different tissue types were exported as a spread-sheet to Excel (Microsoft Office). A two-way analysis of variance (ANOVA), followed by a Benjamini–Hochberg procedure (Benjamini and Hochberg, 1995) to control for false discovery rate (FDR) to counter the effect of multiple testing, were applied in R (version 2.9.2) to identify the buckets/metabolites that differed significantly between the control and the exposed fish. In all instances, FDR < 0.05 was used as the level of significance. Furthermore, in order to investigate whether these buckets also differed significantly between the different exposure groups, a Student’s *t*-test in Excel followed by a Benjamini–Hochberg procedure in R were applied. Also in this case, FDR < 0.05 was applied as the level of statistical significance.

## Results

**Figure 1:**
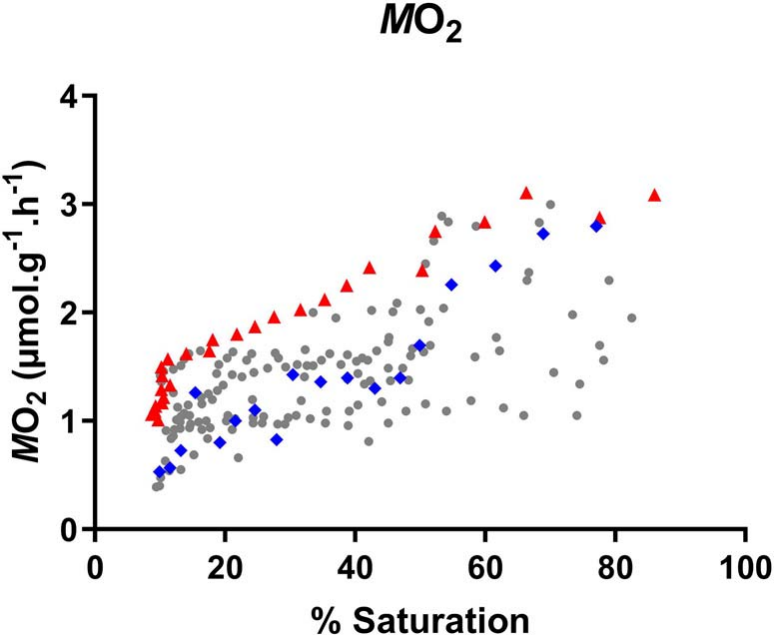
Oxygen consumption rates (*M*O_2_) of spiny dogfish, *Squalus acanthias suckleyi*, during progressive hypoxia in closed respirometry (dots represent *M*O_2_ calculated over 15 min intervals, *N* = 10). Two individual fish with different *M*O_2_ profiles are highlighted with triangles (with apparent critical oxygen level) and diamonds (without apparent critical oxygen level).

In closed respirometry, oxygen consumption rates decreased gradually as hypoxia progressed (*P* < 0.0001, *R*^2^ = 0.3652). In some, but not all of the dogfish, this decrease became faster when hypoxia dropped below approximately 20% saturation (32 Torr). An apparent critical oxygen level (P_crit_) could be calculated on the overall data set by segmented regression analysis (intercept X0 = 18.1% air saturation, 28.5 Torr or 1.6 mg L^−1^) but this gave hardly a better fit (*R*^2^ = 0.3668) compared to the linear regression (*R*^2^ = 0.3652) indicating that this trend for a P_crit_ was not very pronounced. Figure 1 shows the oxygen consumption data, highlighting two individual fish who did or did not show a clear critical oxygen concentration. The fact that there was no clear oxyregulation overall was confirmed when calculating the RI where a hypothetical RI value of 1 indicates perfect regulation of *M*O_2_ down to PO_2_ = 0 while an RI value of 0 indicates perfect conformity (Mueller and Seymour, 2011). Calculated values varied between 0.00 and 0.36 (mean 0.18 ± 0.05).

**Figure 2:**
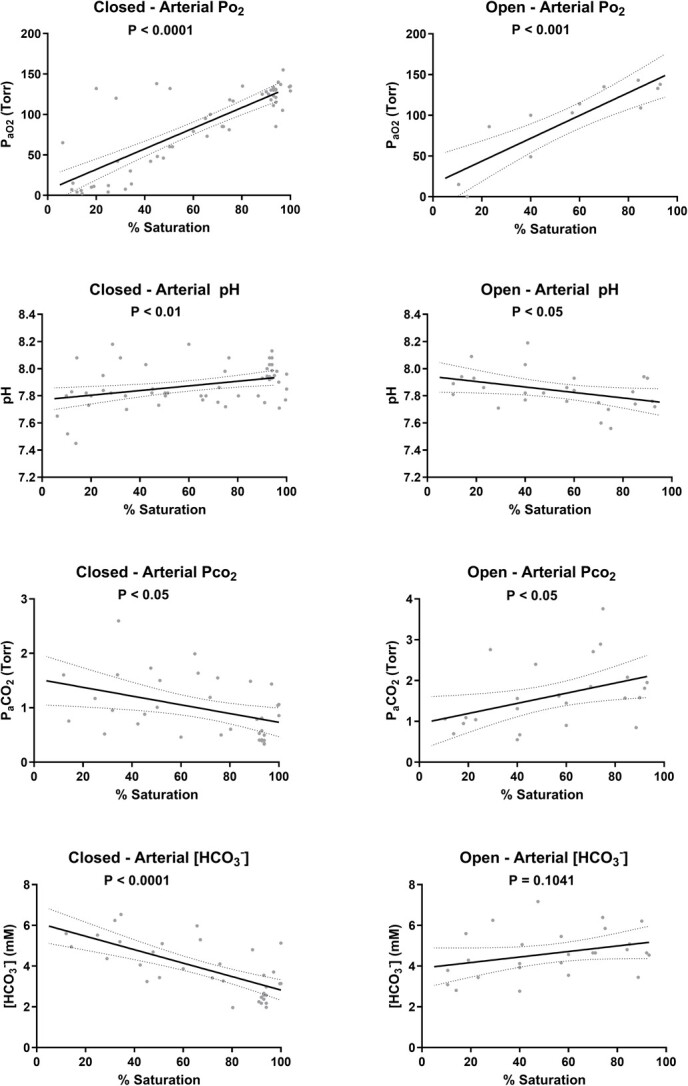
Blood parameters of spiny dogfish, *Squalus acanthias suckleyi*, during progressive hypoxia in closed respirometry (*N* = 5) or open respirometry (*N* = 5) where hypoxia was achieved by N_2_ bubbling. Linear regressions are shown with their 95% confidence intervals and P values are indicated.

**Figure 3:**
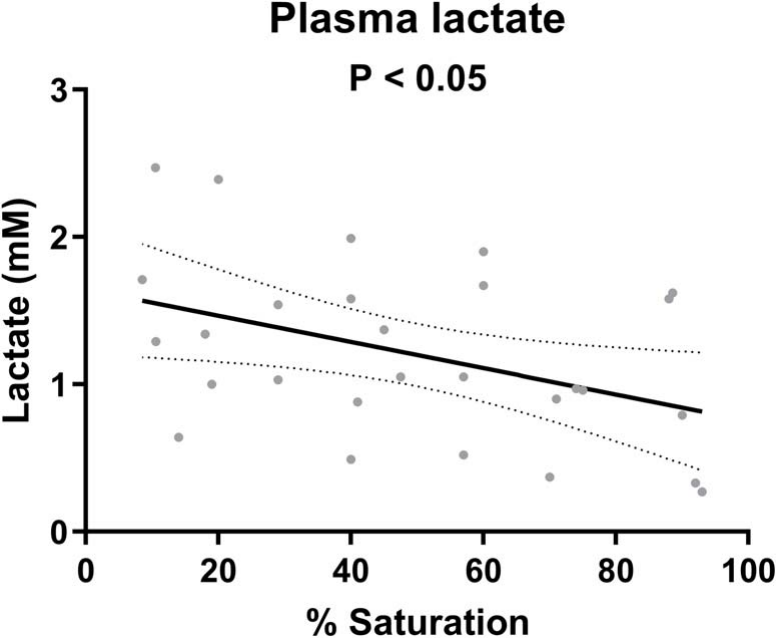
Plasma lactate of spiny dogfish, *Squalus acanthias suckleyi*, during progressive hypoxia in open respirometry (*N* = 5). Linear regression is shown with its 95% confidence intervals (*P* < 0.05).

Regarding arterial blood parameters (Fig. 2), P_a_o_2_ reduced linearly with progressive hypoxia, both under closed respirometry (*P* < 0.0001) and when hypoxia was induced by N_2_ bubbling (*P* < 0.0002). Differences between the two methods were seen in pH_a_, P_a_CO_2_ and [HCO_3_^−^]_a_. In the closed respirometry, where fish were allowed to consume the oxygen present in the box, pH_a_ decreased (*P* < 0.01), P_a_CO_2_ increased (*P* < 0.05) and [HCO_3_^−^]_a_ increased as well (*P* < 0.0001). This was not the case when hypoxia was reached by N_2_ bubbling where pH_a_ increased slightly (*P* < 0.05), P_a_CO_2_ decreased (*P* < 0.05) and [HCO_3_^−^]_a_ did not change significantly_._ In the latter series, lactate was measured and increased significantly (*P* < 0.05) (Fig. 3).

**Figure 4:**
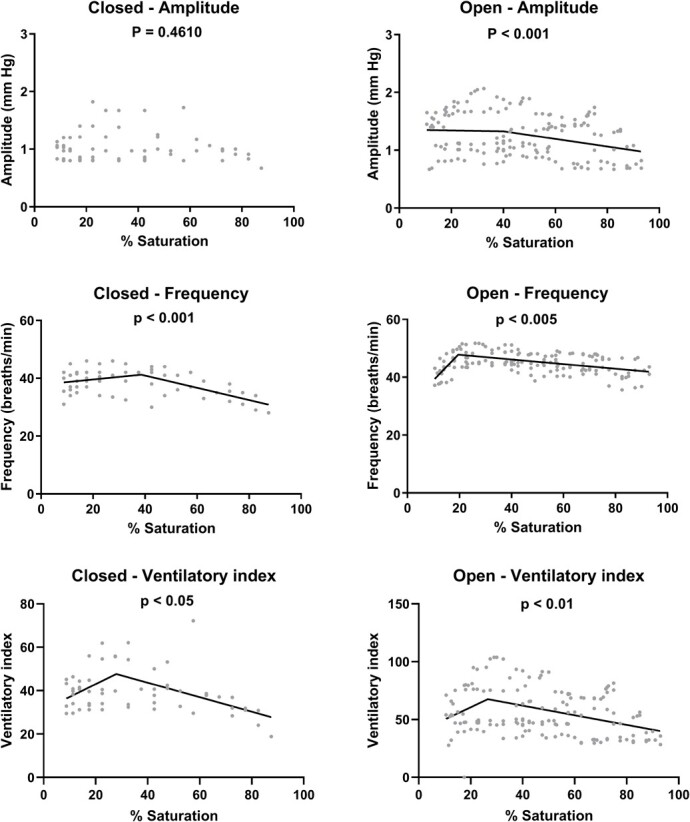
Ventilation amplitude, ventilation frequency and ventilatory index of spiny dogfish, *Squalus acanthias suckleyi*, during progressive hypoxia in closed respirometry (*N* = 5) or open respirometry (*N* = 5) where hypoxia was reached by N_2_ bubbling. *P* values are derived from correlation analysis, segmented linear regression provided a better fit than linear regression and is shown when significant.

Ventilation (Fig. 4) showed significant increases in frequency with decreasing water oxygen levels in both the closed and open system (*P* < 0.001 and *P* < 0.005, respectively), however R^2^ values of both linear fittings were poor (*R*^2^ = 0.1775 and *R*^2^ = 0.0511, respectively). Segmented linear regression showed slightly better fits, with a peak value at 38.5% saturation (60.8 Torr) in the closed system (*R*^2^ = 0.3129), and a peak value at 19.5% saturation (30.8 Torr) in the open system (*R*^2^ = 0.2857). Amplitude showed no significant increase in the closed system, but did show a significant increase with advancing hypoxia in the open system (*P* < 0.001) however with a very poor fitting (*R*^2^ = 0.0782). Segmented linear regression also did not provide a good fit (*R*^2^ < 0.0856). The ventilatory index integrates both frequency and amplitude as a proxy for total ventilatory water flow rate. It significantly increased with progressive hypoxia, both in the closed (*P* < 0.05, *R*^2^ = 0.0691) and open (*P* < 0.01, *R*^2^ = 0.0680) system, both with a better fit when using segmented linear regression (*R*^2^ = 0.2337 and *R*^2^ = 0.1231) and both peaking around 27.5% saturation (43.2 Torr).

Qualitative ^1^H-NMR metabolomics did not provide quantitative data but relative changes compared to controls (Table 1). Only significant changes are shown in Table 1 and discussed. Brain tissue showed no significantly different metabolites between controls and any of the other time-points under hypoxia or recovery. Liver spectral data revealed significantly increased lactate concentrations in extracts of 2-h hypoxic dogfish compared to the control group only. In muscle however, a substantial number of metabolites differentiated in the ^1^H-NMR spectra, and significant differences were seen in isoleucine, valine, β-hydroxybutyrate, lactate, alanine, acetate, glutamate, glutamine, acetoacetate, succinate, carnitine, TMAO/betaine, (phospho)creatine, malonate, taurine, glycine, sarcosine, tartrate, oxypurinol and ATP/ADP/AMP. Hypoxia exposure in general (2 and 6 h) induced decreases in the levels of isoleucine, valine, glutamine, acetoacetate, taurine, glycine, oxypurinol and carnitine (compared to the controls) while the concentrations of lactate, acetate and TMAO increased accordingly. When recovered fish (animals exposed to 6 h of hypoxia and allowed to recover during 6 h of normoxia) were compared to their controls, a recovery was observed for the levels of lactate and acetate but in contrast, recovered fish still displayed lower levels of 3-hydroxybutyrate, isoleucine, valine, glutamine, acetoacetate, taurine, glycine, oxypurinol and carnitine. Similarly, the levels of TMAO were still higher in the recovered group in comparison to the controls.

In gill, ^1^H-NMR analysis of polar extracts also identified a multitude of significant changes. The subsequent buckets/metabolites that were significantly different between control and hypoxic groups were: valine, lactate, glutamate, trimethylamine oxide (TMAO), myo-inositol, (phospho)creatine, phosphocholine, glucose and formate. Both 2 h and 6 h of hypoxic treatment decreased the concentrations of valine, glutamate, myo-inositol, (phospho)creatine, glucose and formate (compared to their respective controls) whereas this was accompanied by increases in the levels of lactate, TMAO and phosphocholine. Furthermore, higher concentrations of lactate, TMAO and phosphocholine were still found in the recovered group associated with lower levels of glutamate, myo-inositol, (phospho)creatine, glucose and formate. Apparently, dogfish did not manage to recover completely from the hypoxic insult (2 and 6 h) during a 6-h reoxygenation since metabolites, which were decreased after hypoxia, were still lower in recovered animals (compared to the controls) and vice versa. Only a recovery for valine levels was observed in dogfish gill extracts.

Rectal gland spectra were investigated by PCA and all significantly different metabolites were found in the 2-h hypoxic group and the recovered group although this was partly caused by the larger variation in the 6-h hypoxia data. Specifically, 2-h hypoxia resulted in increased levels of lactate, taurine, myo-inositol and glycine combined with a decrease in acetate, glutamate and succinate (compared to the controls). Additionally, recovered fish displayed higher levels of taurine and myo-inositol whereas decreases in isoleucine, acetate and were observed (compared to the controls).

## Discussion

The first aim of our study was to determine the hypoxia tolerance of adult Pacific spiny dogfish, *S. suckleyi*, and whether they are oxyregulators or oxyconformers at their natural environmental summer temperature of 12–13°C. Our study shows that the Pacific spiny dogfish tolerated hypoxia relatively well but that they showed large individual variation in their responses. They either behaved as oxygen conformers (pattern C of Wood, 2018: linear relationship between *M*O_2_ and environmental PO_2_) or as oxygen regulators with an apparent P_crit_ around 18% saturation (28.5 Torr, 3.8 kPa or 1.6 mg L^−1^). The latter corresponds most closely to pattern D of Wood (2018) where *M*O_2_ is not maintained during progressive hypoxia but rather falls as external PO_2_ declines, and then falls more acutely below a breakpoint, representing the apparent P_crit_. This apparent P_crit_ value at 18% saturation shows that they are indeed hypoxia tolerant, as it was even lower than in the hypoxia-tolerant epaulette shark *Hemiscyllium ocellatum* which has been assessed to be between 25 and 40% saturation (38.2–60.8 Torr, 5.1–8.1 kPa or 1.6–2.6 mg L^−1^ at 28°C) (Routley *et al*., 2002; Speers-Roesch *et al*., 2012a; Heinrich *et al*., 2014). When kept at about 60% of their P_crit_ at 11–12% air saturation (18 Torr or 1 mg L^−1^) for 6 h, dogfish recovered relatively fast when looking at lactate levels only, consistent with an hypoxia-tolerant phenotype.

The broad spectrum of responses, from oxyconforming to oxyregulating, is not unusual in elasmobranchs. For example in lesser spotted dogfish *Scyliorhinus canicula*, embryos have been seen to be oxyconformers at lower temperatures but oxyregulators at higher temperatures (Thomason *et al*., 1996) and also for adults this seems to be the case (Butler and Taylor, 1975). Also Maugean skate, *Zearaja maugana*, showed to be oxyconforming (Morash *et al*., 2020). A recent review (Waller *et al*., 2024) concludes that the majority of elasmobranchs might be oxyconformers while teleost tend to be oxyregulators (Fritsche and Nilsson, 1993). However, inactive, temperate elasmobranchs can exhibit oxyregulation during hypoxia at lower ambient temperatures, but oxyconform at higher temperatures. This flexible approach on how to handle hypoxia seems to be confirmed by our data, that show high interindividual variation in which strategy is used under environmental hypoxia.

**Table 1:** Significant differences in various metabolite levels based on Area Under the Curve (AUC) intensities between normoxic controls (*N* = 5) and experimental groups: 2 h hypoxia (*N* = 4), 6 h hypoxia (*N* = 4) and 6 h recovery (*N* = 4) (=: no significant difference; ↑: *P* < 0.05; ↑↑: *P* < 0.01; ↑↑↑: *P* < 0.001)

**Tissue**		**Metabolite**	**2 h Hypoxia**	**6 h Hypoxia**	**6 h Recovery**
					
**Brain**			**=**	**=**	**=**
					
**Liver**	Energy metabolism	Lactate	**↑**	**=**	**=**
					
**Muscle**	Energy metabolism	Lactate	**↑↑**	**↑**	**=**
					
	Ketone/Fatty acid	Acetoacetate	**↓↓**	**↓↓**	**↓↓**
		Carnitine	**↓↓**	**↓**	**↓↓**
		β-Hydroxybutyrate	**=**	**=**	**↓**
					
	Amino acids	Alanine			
		Isoleucine	**↓↓**	**↓**	**↓↓**
		Glutamine	**↓↓**	**↓↓↓**	**↓↓**
		Glycine	**↓↓**	**↓**	**↓↓**
		Taurine	**↓↓**	**↓**	**↓↓**
		Valine	**↓↓**	**↓**	**↓↓**
					
	Other	Acetate	**↑**	**=**	**=**
		Oxypurinol	**↓↓**	**↓↓**	**↓**
		Trimethylamineoxide/betaine	**↑↑**	**↑**	**↑↑**
					
**Gill**	Energy metabolism	Lactate	**↑↑**	**↑↑**	**↑**
		Glucose	**↓↓**	**↓↓**	**↓↓**
		(Phospho)creatine	**↓↓**	**↓↓**	**↓↓**
					
	Amino acids	Glutamate	**↓↓**	**↓↓**	**↓↓**
		Valine	**↓**	**↓**	**=**
					
	Other	Formate	**↓↓**	**↓↓**	**↓↓**
		Myo-inositol	**↓↓**	**↓↓**	**↓↓**
		Phosphocholine	**↑↑**	**↑↑**	**↑↑**
		Trimethylamineoxide	**↑↑**	**↑↑**	**↑↑**
					
**Rectal gland**	Energy metabolism	Lactate	**↑↑↑**	**=**	**=**
					
	Ketone/Fatty acid	Succinate	**↓↓↓**	**=**	**=**

**Table 1:** Continued

**Tissue**		**Metabolite**	**2 h Hypoxia**	**6 h Hypoxia**	**6 h Recovery**
					
	Amino acids	Isoleucine	**=**	**=**	**↓↓↓**
		Glutamate	**↓↓↓**	**=**	**↓**
		Glycine	**↑**	**=**	**=**
		Taurine	**↑↑**	**=**	**↑↑**
					
	Other	Acetate	**↓↓**	**=**	**↓↓↓**
		Myo-inositol	**↑↑**	**=**	**↑↑**
					

In adult Pacific spiny dogfish, previous reports indicate that both ventilation amplitude and frequency increased significantly (Perry and Gilmour, 1996; Acharya-Patel *et al*., 2018). We predicted that internal hypoxia (arterial changes in blood gasses, acid–base regulation and anaerobic metabolites) would precede changes in ventilation and P_crit_. Our study showed a significant increase in ventilation frequency in the closed system, where P_crit_ was measured, and an increase in both frequency and amplitude in the open system, leading to a peak in ventilatory index in both systems. The peak of hyperventilation occurred earlier (38.5% air saturation or 60.8 Torr) and well before P_crit_ in the closed system compared to the open (19.5% air saturation or 30.8 Torr) system which most likely can be explained by the build-up of carbon dioxide in the system (Perry and Gilmour, 2002). Hypercarbia does induce hyperventilation in Pacific spiny dogfish (Perry and Gilmour, 1996) and was evident from the increased plasma P_a_CO_2_ and the decreased blood pH_a_ in the fish in the closed system. Current evidence indicates that branchial CO_2_/pH chemoreceptors are expected to respond to external rather than internal increases in CO_2_ levels in Pacific spiny dogfish (McKendry *et al*., 2001; Perry and McKendry, 2001), but in this case both external and internal hypercarbia likely occurred. The earlier onset of hyperventilation in the closed system is therefore most likely triggered by increased CO_2_ rather than reduced O_2_. It can be discussed whether one should use closed or open respirometry during P_crit_ measurements (Rogers *et al*., 2016), but to our opinion, this depends on the research question. Open respirometry isolates the effects of reducing oxygen levels alone, but closed respirometry might be a better representation of natural hypoxia, as oxygen poor environments are often rich in CO_2_. Our aim was to assess how the Pacific spiny dogfish would cope with hypoxia in their natural environment. As a benthic species, they would most likely encounter a combination of low O_2_ and high CO_2_ and therefore closed respirometry seems most appropriate.

It is clear that the dogfish attempted to regulate gas exchange by increasing ventilation near the P_crit_ (28.5 Torr, 3.8 kPa or 1.6 mg L^−1^), and some dogfish clearly managed better than others with P_a_o_2_ levels remaining well above the average regression line under hypoxic conditions. Overall, this regulation, although clearly present, remained weak as the calculated RI varied between 0.00 (perfect conformer) and 0.36, indicating limitations to regulate oxygen consumption rates when environmental hypoxia occurs. Hyperventilation responses have been seen in other shark species as well, e.g. bonnetheads increased mouth gape and ventilation volume (Carlson and Parsons, 2003), and epaulette shark increased their ventilatory rate (Routley *et al*., 2002) which allowed them to successfully maintain *M*O_2_. These responses could come at a cost, as it was suggested that severe hypoxia (5% air saturation or 8 Torr) in dogfish can not only lead to a reduction in active gill homeostatic processes, such as urea retention, acid–base regulation and ionoregulation but also induce an osmoregulatory compromise because of increased functional gill surface area and water flow (Zimmer and Wood, 2014). In the same study at 20% air saturation (31.5 Torr), just above the observed P_crit_, effects were limited. As in the epaulette shark (Routley *et al*., 2002; Speers-Roesch *et al*., 2012a), lactate production was only significant at and below the P_crit_. Similarly, arterial P_a_o_2_ decreased linearly with progressive hypoxia in both our study with spiny dogfish and in epaulette shark (Speers-Roesch *et al*., 2012a). As P_crit_ and haemoglobin–O_2_ saturation are said to be strongly linked (Mandic *et al*., 2009; Speers-Roesch *et al*., 2012a; Morrison *et al*., 2016), the low P_crit_ also suggests a high binding capacity of *S. suckleyi* haemoglobin. However, measured Oxygen-Hb dissociation curves on whole blood give blood oxygen affinities or 50% saturation levels (P_50_) at 17 Torr (Lenfant and Johansen, 1966), which is not considered exceptionally low. When comparing over several elasmobranch species, a broad range of of P_50_ and P_crit_ values is seen (P_50_ 14–47 Torr, P_crit_ 25–70% air saturation), but not necessarily a strict correlation between the two (Brill and Lai, 2016).

Elasmobranchs show an unusual metabolism compared to teleosts and all other vertebrates (Speers-Roesch and Treberg, 2010). Whereas all tissues show normal carbohydrate metabolism, muscle and heart have only limited or no fatty acid oxidation and show a great reliance on ketone bodies (e.g. ß-hydroxybutyrate) and amino acids as oxidative fuels. Other tissues do have a significant capacity to oxidize fatty acids but also rely on ketone bodies and amino acids. The liver not only represents the lipid storage site and acts as a buoyancy device, its amino acid- and lipid fueled ketogenesis sustains the demand for ketone bodies in other tissues (Ballantyne and Speers-Roesch, 2006; Speers-Roesch and Treberg, 2010). In our study, hypoxic treatment resulted in significant changes in a range of metabolites in the spiny dogfish but overall, most changes were found in white muscle tissue, one of the two tissues that heavily relies on ketones. Regrettably, we did not sample heart tissue, so we cannot confirm whether this is linked to their lack of fatty acid oxidation.

When dogfish were exposed to hypoxia (2 and 6 h) and allowed to recover (6 h) under normoxia, no significant changes were found in brain metabolite levels. It is difficult to tell whether the absence of significant brain metabolite changes following hypoxia is due to a sufficient energy supply (from own or from other tissues) or possibly due to metabolic depression in brain activity. In liver, only lactate levels showed significant increases at the onset of the hypoxic insult, after which they recovered. The same trend was seen in rectal gland. In muscle and gill tissue, lactate levels were increased for the entire duration hypoxic period. While they recovered after 6 h of reoxygenation in muscle, they did not in gill tissue. In elasmobranchs, lactate concentrations are known to rise quickly when they are exposed to acute stress such as diminished oxygen levels (Renshaw and Chapman, 2009) indicating significant up-regulation of anaerobic energy metabolism. Glucose is released in elasmobranchs as an energy source in response to stress (Anderson, 2012; Bouyoucos *et al*., 2018; Lambert *et al*., 2018; Ruiz-Jarabo *et al*., 2019; Schoen *et al*., 2021) but glucose and phosphocreatine levels were surprisingly unaffected (except in the gills), similar to the results of previous studies on other elasmobranchs exposed to hypoxia (Routley *et al*., 2002; Speers-Roesch and Treberg, 2010; Speers-Roesch *et al*., 2012b).

The reliance on ketone bodies was demonstrated by the decreased concentrations of acetoacetate in white muscle. Both acetoacetate and β-hydroxybutyrate are generated during metabolism of fatty acid oxidation. In mammals, they mostly serve as metabolic fuels in extrahepatic tissues during fasting, but elasmobranchs continuously rely on these ketone bodies to power heart and muscle tissues. In contrast to glucose and lactate, levels of acetoacetate remained reduced after the recovery period. β-hydroxybutyrate levels were not reduced during hypoxia, but levels dropped during recovery, perhaps in the process of replenishing acetoacetate levels. These results correspond well with other studies showing that elasmobranch muscles do not rely on lipid oxidation but, instead, prefer to use ketone bodies as major oxidative fuels (Zammit and Newsholme, 1979; Moyes *et al*., 1990; Ballantyne *et al*., 1992; Ballantyne and Speers-Roesch, 2006). The latter is in sharp contrast to the situation in teleosts where red muscle and heart heavily rely on fatty acid oxidation (Ballantyne and Speers-Roesch, 2006). Our study actually found a decrease in carnitine in white muscle during hypoxia and recovery. Carnitine facilitates the transport of fatty acids into the mitochondria, indicating a reduced importance of fatty acid oxidation. This process is also involved in the production of ketones such as acetoacetate and β-hydroxybutyrate, which were also reduced during hypoxia and/or recovery. Furthermore, amino acids were heavily used in white muscle and decreases of alanine, isoleucine, glutamine, glycine, taurine and valine were observed. None of these recovered during the 6-h recovery period.

Other changes indicate osmoregulatory disturbance. Reduced glutamate in the two most important osmoregulatory organs, rectal gland and gills and of glutamine in white muscle, might indicate changes in urea metabolism. Indeed, exposure to severe hypoxia leads to a significant increase in urea excretion, suggesting a loss of urea retention (Zimmer and Wood, 2014). Further indications of compromised osmoregulation are the increase of trimethylamine oxide (TMAO) in gills and muscle. Elasmobranchs typically possess substantial concentrations of methylamines in their tissues, particularly TMAO, glycine and betaine in order to counter the potentially denaturing consequences of characteristically high urea concentrations in the blood and tissues of these animals (Yancey and Somero, 1979; Seibel and Walsh, 2002). Increased taurine levels in the rectal gland could also be seen as a possible osmoregulatory response, or as a protective mechanism against oxidative stress. Taurine is an organic osmolyte involved in cell volume regulation, plays a role in the modulation of intracellular free calcium concentration, and is also a powerful antioxidant in fish (Ceccotti *et al*., 2019). Other indications of oxidative stress could be the decrease of oxypurinol in muscle. Oxypurinol is the active metabolite of allopurinol and a xanthine oxidase inhibitor, hereby inhibiting the enzymatic generation of reactive oxygen species by xanthine oxidase. Although allopurinol is perfectly suited to scavenge highly reactive hydroxyl radicals, oxypurinol performs this important task more effectively than is allopurinol. Moreover, in the present context of tissue hypoxia and possibly reoxygenation injury, oxypurinol might play an important role in protecting tissues and cells against the detrimental effects of reperfusion damage after hypoxic insults (Halliwell *et al*., 1987).

In conclusion, we show evidence that *Squalus suckleyi* is a hypoxia tolerant species, which through hyperventilation maintains a slowly decreasing but reasonable *M*O_2_ down to 18.1% air saturation (28.5 Torr) after which *M*O_2_ declines very quickly. Nevertheless, individual strategies seem to vary and a P_crit_ is not clearly present in each individual. When exposing the Pacific spiny dogfish for 6 hours to hypoxia at 11–12% air saturation (18 Torr), about 60% of the critical oxygen level, the brain is still extremely well protected from the hypoxic insult as no metabolic changes were observed in this tissue. Possibly an efficient lactate metabolism could play a role here. The importance of ketone bodies and amino acids in metabolism of white muscle in elasmobranchs was confirmed. Hypoxia not only affected both aerobic and anaerobic metabolism, but based on a previous study (Zimmer and Wood, 2014) also appeared to interfere with osmoregulation possibly through the osmorespiratory compromise. Indeed, hyperventilation for oxyregulation can at the same time induce increased ion- and waterfluxes such as urea loss (Zimmer and Wood, 2014). Indications of disturbed osmoregulation and oxidative stress could be found in changes of molecules such as TMAO and other protective compounds such as myo-inositol, taurine and oxypurinol. Finally, when only looking at classic indicators of metabolic stress, such as glucose and lactate, it could be concluded that the dogfish fully recovered after 6 hours of reoxygention. However, the persistent changes in ketones, amino acids and other compounds proved otherwise.

Overall, our data show that adult *S. suckleyi* show a remarkable variation in response to hypoxia, from oxyregulation to osmoconforming. Furthermore, we demonstrated that Pacific spiny dogfish can tolerate transient episodes of severe hypoxia, and can regulate their oxygen consumption rates down to low environmental oxygen levels (low P_crit_). However, recovery from such an hypoxic insult takes a long time suggesting that more frequent and longer periods of coastal hypoxia could compromise their chances of survival in the future. This long recovery would not be picked up by measurement of traditional biomarkers such as the occurrence of increased lactate levels, indicating the importance of using the appropriate biomarkers in conservation management.

## Data Availability

The data used in this article is available on request to the corresponding author.

## References

[ref1] Acharya-Patel N , Deck CA, Milsom WK (2018) Cardiorespiratory interactions in the Pacific spiny dogfish, *Squalus suckleyi*. J Exp Biol 221: jeb183830. 10.1242/jeb.183830. 30012576

[ref2] Anderson WG (2012) The endocrinology of 1α -hydroxycorticosterone in elasmobranch fish: A review. Comp Biochem Physiol A Mol Integr Physiol 162: 73–80. 10.1016/j.cbpa.2011.08.015. 21911073

[ref3] Ballantyne JS (1997) Jaws: the inside story. The metabolism of elasmobranch fishes. Comp Biochem Physiol 118: 703–742. 10.1016/S0305-0491(97)00272-1.

[ref4] Ballantyne JS , Chamberlin ME, Singer TD (1992) Oxidative metabolism in thermogenic tissues of the swordfish and mako shark. J Exp Zool 261: 110–114. 10.1002/jez.1402610113.

[ref5] Ballantyne JS , Speers-Roesch B (2006) Metabolic organization of freshwater, euryhaline, and marine elasmobranchs: implications for the evolution of energy metabolism in sharks and rays. J Exp Biol 209: 2495–2508. 10.1242/jeb.02294. 16788033

[ref6] Benjamini Y , Hochberg Y (1995) Controlling the false discovery rate: a practical and powerful approach to multiple testing. J R Stat Soc B 57: 289–300. 10.1111/j.2517-6161.1995.tb02031.x.

[ref7] Bindoff NL , Cheung WWL, Kairo JG, Arístegui J, Guinder VA, Hallberg R, Hilmi N, Jiao N, Karim MS, Levin L et al. (2019) Changing ocean, marine ecosystems, and dependent communities. In HO Pörtner, DC Roberts, V Masson-Delmotte, P Zhai, M Tignor, E Poloczanska, K Mintenbeck, A Alegría, M Nicolai, A Okem et al., eds, IPCC Special Report on the Ocean and Cryosphere in a Changing Climate. Cambridge University Press, Cambridge, UK and New York, pp. 447–587

[ref77] Boutilier RG , Heming TA, Iwama GK (1984) Appendix: physicochemical parameters for use in fish respiratory physiology. In: WS Hoar, DJ Randall, eds, *Fish Physiology*, vol 10A. Academic Press, Cambridge, pp. 403–430. 10.1016/S1546-5098(08)60323-4.

[ref8] Bouyoucos IA , Weideli OC, Planes S, Simpfendorfer CA, Rummer JL (2018) Dead tired: evaluating the physiological status and survival of neonatal reef sharks under stress. Conserv Physiol 6: coy053. 10.1093/conphys/coy053. 30254751 PMC6142904

[ref9] Breitburg D , Levin LA, Oschlies A, Grégoire M, Chavez FP, Conley DJ, Garçon V, Gilbert D, Gutiérrez D, Isensee K et al. (2018) Declining oxygen in the global ocean and coastal waters. Science 359: eaam7240. 10.1126/science.aam7240. 29301986

[ref10] Brill RW , Lai NC (2016) Elasmobranch cardiovascular system. In RE Shadwick, AP Farrell, CJ Brauner, eds, Physiology of Elasmobranch Fishes: Internal Processes, Fish Physiology 34B. Elsevier BV, Amsterdam, pp. 1–82

[ref11] Butler PJ , Taylor EW (1975) The effect of progressive hypoxia on respiration in the dogfish (*Scyliorhinus canicula*) at different seasonal temperatures. J Exp Biol 63: 117–130. 10.1242/jeb.63.1.117. 1159356

[ref12] Carlson JK , Parsons GR (2003) Respiratory and hematological responses of the bonnethead shark, *Sphyrna tiburo*, to acute changes in dissolved oxygen. J Exp Mar Biol Ecol 294: 15–26. 10.1016/S0022-0981(03)00237-5.

[ref13] Carstensen J , Andersen JH, Gustafsson BG, Conley DJ (2014) Deoxygenation of the Baltic Sea during the last century. Proc Natl Acad Sci USA 111: 5628–5633. 10.1073/pnas.1323156111. 24706804 PMC3992700

[ref14] Ceccotti C , Al-Sulaivany BSA, Al-Habbib OAM, Saroglia M, Rimoldi S, Terova G (2019) Protective effect of dietary taurine from ROS production in European Seabass under Conditions of Forced Swimming. Animals 9: 607. 10.3390/ani9090607. 31454952 PMC6770007

[ref15] Chamberlin ME , Ballantyne JS (1992) Glutamine metabolism in elasmobranch and agnathan muscle. J Exp Zool 264: 267–272. 10.1002/jez.1402640306.

[ref16] Chan DKO , Wong TM (1977) Physiological adjustments to dilution of the external medium in the lip shark, *Hemiscyllium plagiosum* (Bennet). III Oxygen consumption and metabolic rates. J Exp Zool 200: 97–102. 10.1002/jez.1402000112.

[ref17] Compagno LJV (1990) Alternative life-history styles of cartilaginous fishes in time and space. Environ Biol Fishes 28: 33–75. 10.1007/BF00751027.

[ref18] De Boeck G , Wood CM (2015) Does ammonia trigger ventilation in the dogfish shark, *Squalus acanthias suckleyi*? Respir Physiol Neurobiol 206: 25–35. 10.1016/j.resp.2014.11.009. 25462837

[ref19] Dejours P (1981) Principles of Comparative Respiratory Physiology. Elsevier/North-Holland Biomedical Press, Amsterdam, New York, Oxford

[ref20] Dulvy NK , Pacoureau N, Rigby CL, Pollom RA, Jabado RW, Ebert DA, Finucci B, Pollock CM, Cheok J, Derrick DH et al. (2021) Overfishing drives over one-third of all sharks and rays toward a global extinction crisis. Curr Biol 31: 5118–5119. 10.1016/j.cub.2021.11.008. 34813743

[ref21] Ebert DA (2003) Sharks, rays and chimaeras of California. California Natural History Guides No. 71. University of California Press, Oakland

[ref22] Fan TWM (1996) Metabolite profiling by one- and two-dimensional NMR analysis of complex mixtures. Prog Nucl Magn Reson Spectrosc 28: 161–219. 10.1016/0079-6565(95)01017-3.

[ref23] Farrell AP , Mueller CA, Seymour RS (2021) Coming up for air. J Exp Biol 224: jeb243101. 10.1242/jeb.243101. 34522951

[ref24] Ferretti F , Worm B, Britten GL, Heithaus MR, Lotze HK (2010) Patterns and ecosystem consequences of shark declines in the ocean. Ecol Lett 13: 1055–1071. 10.1111/j.1461-0248.2010.01489.x. 20528897

[ref25] Fritsche R , Nilsson S (1993) Cardiovascular and ventilatory control during hypoxia. In JC Rankin, FB Jensen, eds, Fish Ecophysiology. Chapman & Hall Fish and Fisheries Series, vol 9. Springer, Dordrecht, pp. 180–206

[ref26] Giacomin M , Schulte P, Wood CM (2017) Differential effects of temperature on oxygen consumption and branchial fluxes of urea, ammonia and water in the dogfish shark (*Squalus acanthias suckleyi*). Physiol Biochem Zool 90: 627–637. 10.1086/694296. 28972451

[ref27] Giacomin M , Schulte P, Wood CM (2022) Osmoregulatory compromise in an elasmobranch: oxygen consumption, ventilation and nitrogen metabolism during recovery from exhaustive exercise in dogfish sharks (*Squalus suckleyii*). J Comp Physiol B 192: 647–657. 10.1007/s00360-022-01447-4. 35838789

[ref28] Gobler CJ , Baumann H (2016) Hypoxia and acidification in ocean ecosystems: coupled dynamics and effects on marine life. Biol Lett 12: 20150976. 10.1098/rsbl.2015.0976. 27146441 PMC4892234

[ref29] Govindaraju V , Young K, Maudsley AA (2000) Proton NMR chemical shifts and coupling constants for brain metabolites. NMR Biomed 13: 129–153. 10.1002/1099-1492(200005)13:3<129::AID-NBM619>3.0.CO;2-V. 10861994

[ref30] Halliwell B , Moorhouse PC, Grootveld M, Quinlan JG, Gutteridge JMC (1987) Allopurinol and oxypurinol are hydroxyl radical scavengers. FEBS J 213: 23–28. 10.1016/0014-5793(87)81458-8. 3030809

[ref31] Heinrich DD , Rummer JL, Morash AJ, Watson SA, Simpfendorfer CA, Heupel MR, Munday PL (2014) A product of its environment: the epaulette shark (*Hemiscyllium ocellatum*) exhibits physiological tolerance to elevated environmental CO_2_. Conserv Physiol 2: cou047. 10.1093/conphys/cou047. 27293668 PMC4806737

[ref32] Intergovernmental Oceanographic Commission (2018) The ocean is losing its breath: Declining oxygen in the world’s ocean and coastal waters. In D Breitburg, M Gregoire, K Isensee, eds, IOC Technical Series No. 137 (IOC/2018/TS/137). Intergovernmental Oceanographic Commission of UNESCO (IOC-UNESCO), Paris, 40pp

[ref33] IPCC (2019) In HO Pörtner, DC Roberts, V Masson-Delmotte, P Zhai, M Tignor, E Poloczanska, K Mintenbeck, A Alegría, M Nicolai, A Okem et al., eds, IPCC Special Report on the Ocean and Cryosphere in a Changing Climate. Cambridge University Press, Cambridge, UK and New York, 755 pp

[ref34] Lambert FN , Treberg JR, Anderson WG, Brandt C, Evans AN (2018) The physiological stress response of the Atlantic stingray (*Hypanus sabinus*) to aerial exposure. Comp Biochem Physiol 219-220: 38–43. 10.1016/j.cbpa.2018.02.009. 29482030

[ref35] Lardon I , Eyckmans M, Vu TN, Laukens K, De Boeck G, Dommisse R (2013) ^1^H-NMR study of the metabolome of a moderately hypoxia-tolerant fish, the common carp (*Cyprinus carpio*). Metabolomisc 9: 1216–1227. 10.1007/s11306-013-0540-y.

[ref36] Lardon I , Nilsson GE, Stecyk JAW, Vu TN, Laukens K, Dommisse R, De Boeck G (2012) ^1^H-NMR study of the metabolome of an exceptionally anoxia-tolerant vertebrate, the crucian carp (*Carassius carassius*). Metabolomics 9: 311–323. 10.1007/s11306-012-0448-y.

[ref37] Lenfant C , Johansen K (1966) Respiratory function in the elasmobranch *Squalus suckleyi* G. Resp Physiol 1: 13–29. 10.1016/0034-5687(66)90025-9. 5912135

[ref38] Lin CY , Wu HF, Tjeerdema RS, Viant MR (2007) Evaluation of metabolite extraction strategies from tissue samples using NMR metabolomics. Metabolomics 3: 55–67. 10.1007/s11306-006-0043-1.

[ref39] Lindon JC , Nicholson JK, Holmes E (2007) Handbook of Metabonomics and Metabolomics. Elsevier, Amsterdam, p. 572

[ref40] MacNeil MA , Chapman DD, Heupel M, Simpfendorfer CA, Heithaus M, Meekan M, Harvey E, Goetze J, Kiszka J, Bond ME et al. (2020) Global status and conservation potential of reef sharks. Nature 583: 801–806. 10.1038/s41586-020-2519-y. 32699418

[ref41] Mandic M , Todgham AE, Richards JG (2009) Mechanisms and evolution of hypoxia tolerance in fish. Proc R Soc B 276: 735–744. 10.1098/rspb.2008.1235. PMC2660936 18996831

[ref42] McKendry JE , Milsom WK, Perry SF (2001) Branchial CO_2_receptors and cardiorespiratory adjustments during hypercarbia in Pacific spiny dogfish (*Squalus acanthias*). J Exp Biol 204: 1519–1527. 10.1242/jeb.204.8.1519. 11273813

[ref43] Metcalfe JD , Butler PJ (1984) Changes in activity and ventilation response to hypoxia in unrestrained, unoperated dogfish, *Scyliorhinus canicula*. J Expl Biol 108: 411–418. 10.1242/jeb.108.1.411. 6707572

[ref44] Milsom WK , Taylor EW (2016) Control of breathing in elasmobranchs. In RE Shadwick, AP Farrell, CJ Brauner, eds, Physiology of Elasmobranch Fishes: Internal Processes. Academic Press, Cambridge, pp. 83–126

[ref45] Morash AJ , Lyle JM, Currie S, Bell JD, Stehfest KM, Semmens JM (2020) The endemic and endangered Maugean Skate (*Zearaja maugeana*) exhibits short-term severe hypoxia tolerance. Conserv Physiol 8: coz105. 10.1093/conphys/coz105. 31976076 PMC6969080

[ref46] Morrison PR , Gilmour KM, Brauner CJ (2016) Oxygen and carbon dioxide transport in elasmobranchs. In RE Shadwick, AP Farrell, CJ Brauner, eds, Physiology of Elasmobranch Fishes: Internal Processes. Academic Press, Cambridge, pp. 127–219

[ref47] Moyes CD , Buck LT, Hochachka PW (1990) Mitochondrial and peroxisomal fatty acid oxidation in elasmobranchs. Am J Physiol 258: R756–R762. 10.1152/ajpregu.1990.258.3.R756. 2316720

[ref48] Mueller CA , Seymour RS (2011) The regulation index: a new method for assessing the relationship between oxygen consumption and environmental oxygen. Physiol Biochem Zool 84: 522–532. 10.1086/661953. 21897089

[ref49] Pacoureau N , Rigby CL, Kyne PM, Sherley RB, Winker H, Carlson JK, Fordham SV, Barreto R, Fernando D, Francis MP et al. (2021) Half a century of global decline in oceanic sharks and rays. Nature 589: 567–571. 10.1038/s41586-020-03173-9. 33505035

[ref50] Perry SF , Gilmour KM (1996) Consequences of catecholamine release on ventilation and blood oxygen transport during hypoxia and hypercapnia in an elasmobranchs (*Squalus acanthias*) and a teleost (*Oncorhynchus mykiss*). J Exp Biol 199: 2105–2118. 10.1242/jeb.199.9.2105. 9320017

[ref51] Perry SF , Gilmour KM (2002) Sensing and transfer of respiratory gases at the fish gill. J Exp Zool 293: 249–263. 10.1002/jez.10129. 12115900

[ref52] Perry SF , McKendry JE (2001) The relative roles of external and internal CO_2_ versus H^+^ in eliciting the cardiorespiratory responses of *Salmo salar* and *Squalus acanthias* to hypercarbia. J Exp Biol 204: 3963–3971. 10.1242/jeb.204.22.3963. 11807114

[ref53] Piiper J , Baumgarten D, Meyer M (1970) Effects of hypoxia upon respiration and circulation in the dogfish *Scyliorhinus stellaris*. Comp Biochem Physiol 36: 513–520. 10.1016/0010-406X(70)91027-3. 5475905

[ref54] Rabalais NN , Díaz RJ, Levin LA, Turner RE, Gilbert D, Zhang J (2010) Dynamics and distribution of natural and human-caused hypoxia. Biogeosciences 7: 585–619. 10.5194/bg-7-585-2010.

[ref55] Regan MD , Mandic M, Dhillon RS, Lau GY, Farrell AP, Schulte PM, Seibel BA, Speers-Roesch B, Ultsch GR, Richards JG (2019) Don't throw the fish out with the respirometry water. J Exp Biol 222: jeb200253. 10.1242/jeb.200253. 30923073

[ref56] Renshaw GMC , Chapman CA (2009) Hematological responses of the grey carpet shark (*Chiloscyllium punctatum*) and the epaulette shark (*Hemiscyllium ocellatum*) to anoxia and reoxygenation. J Exp Zool 311: 422–438. 10.1002/jez.539 19405134

[ref57] Roff G , Doropoulos C, Rogers A, Bozec YM, Krueck NC, Aurellado E, Priest M, Birrell C, Mumby PJ (2016) The ecological role of sharks on coral reefs. Trends Ecol Evol 31: 395–407. 10.1016/j.tree.2016.02.014. 26975420

[ref58] Rogers NJ , Urbina MA, Reardon EE, McKenzie DJ, Wilson RW (2016) A new analysis of hypoxia tolerance in fishes using a database of critical oxygen level (P_crit_). Conserv Physiol 4: cow012. 10.1093/conphys/cow012. 27293760 PMC4849809

[ref59] Routley MH , Nilsson GE, Renshaw GMC (2002) Exposure to hypoxia primes the respiratory and metabolic responses of the epaulette shark to progressive hypoxia. Comp Biochem Physiol A Mol Integr Physiol 131: 313–321. 10.1016/s1095-6433(01)00484-6. 11818221

[ref60] Ruiz-Jarabo I , Berragan-Mendez C, Jerez-Cepa I, Fernandez-Castro M, Sobrino I, Mancera JM, Aerts J (2019) Plasma 1α-Hydroxycorticosterone as biomarker for acute stress in catsharks (*Scyliorhinus canicula*). Front Physiol 10: 1217. 10.3389/fphys.2019.01217. 31616315 PMC6764463

[ref61] Schoen AN , Treberg JR, Wheaton CJ, Mylniczenko N, Anderson WG (2021) Energy and corticosteroid mobilization following an induced stress response in an elasmobranch fish, the North Pacific spiny dogfish (*Squalus acanthias suckleyi*). Gen Comp Endocrinol 310: 113799. 10.1016/j.ygcen.2021.113799. 33961877

[ref62] Seibel BA , Walsh PJ (2002) Trimethylamine oxide accumulation in marine animals: relationship to acylglycerol storage. J Exp Biol 205: 297–306. 10.1242/jeb.205.3.297. 11854367

[ref64] Speers-Roesch B , Brauner CJ, Farrell AP, Hickey AJR, Renshaw GMC, Wang YS, Richards JG (2012b) Hypoxia tolerance in elasmobranchs. II. Cardiovascular function and tissue metabolic responses during progressive and relative hypoxia exposures. J Exp Biol 215: 103–114. 10.1242/jeb.059667. 22162858

[ref65] Speers-Roesch B , Mandic M, Groom DJE, Richards JG (2013) Critical oxygen tensions as predictors of hypoxia tolerance and tissue metabolic responses during hypoxia exposure in fishes. J Exp Mar Biol Ecol 449: 239–249. 10.1016/j.jembe.2013.10.006.

[ref66] Speers-Roesch B , Richards JG, Brauner CJ, Farrell AP, Hickey AJR, Wang YS, Renshaw GMC (2012a) Hypoxia tolerance in elasmobranchs. I. Critical oxygen tension as a measure of blood oxygen transport during hypoxia exposure. J Exp Biol 215: 93–102. 10.1242/jeb.059642. 22162857

[ref67] Speers-Roesch B , Treberg JR (2010) The unusual energy metabolism of elasmobranch fishes. Comp Biochem Physiol A Mol Integr Physiol 155: 417–434. 10.1016/j.cbpa.2009.09.031. 19822221

[ref68] Thomason JC , Davenport J, Le Comte E (1996) Ventilatory mechanisms and the effect of hypoxia and temperature on the embryonic lesser spotted dogfish. J Fish Biol 49: 965–972. 10.1111/j.1095-8649.1996.tb00093.x.

[ref69] Waller MJ , Humphries NE, Womersley FC, Loveridge A, Jeffries AL, Watanabe Y, Payne N, Semmens J, Queiroz N, Southall EJ et al. (2024) The vulnerability of sharks, skates, and rays to ocean deoxygenation: Physiological mechanisms, behavioral responses, and ecological impacts. J Fish Biol 1–30. 10.1111/jfb.1583030. 38852616

[ref70] Wood CM (2018) The fallacy of the P_crit_ – are there more useful alternatives? J Exp Biol 221: jeb163717. 10.1242/jeb.163717. 30420494

[ref71] Wood CM , Bucking CP, Fitzpatrick J, Nadella SR (2007) The alkaline tide goes out and the nitrogen stays in after feeding in the dogfish shark, *Squalus acanthias*. Respir Physiol Neurobiol 159: 163–170. 10.1016/j.resp.2007.06.008. 17656159

[ref72] Wootton TP , Sepulveda CA, Wegner NC (2015) Gill morphometrics of the thresher sharks (genus Alopias): correlation of gill dimensions with aerobic demand and environmental oxygen. J Morphol 276: 589–600. 10.1002/jmor.20369. 25703507

[ref73] Wu HF , Southam AD, Hines A, Viant MR (2008) High-throughput tissue extraction protocol for NMR and MS-based metabolomics. J Anal Biochem 372: 204–212. 10.1016/j.ab.2007.10.002. 17963684

[ref74] Yancey PH , Somero GN (1979) Counteraction of urea destabilisation of protein structure by methylamine osmoregulatory compounds of elasmobranchs fishes. J Biochem 183: 317–323. 10.1042/bj1830317. PMC1161561 534499

[ref75] Zammit VA , Newsholme EA (1979) Activities of enzymes of fat and ketone-body metabolism and effects of starvation on blood concentrations of glucose and fat fuels in teleost and elasmobranch fish. J Biochem 184: 313–322. 10.1042/bj1840313. PMC1161766 534530

[ref76] Zimmer AM , Wood CM (2014) Exposure to acute severe hypoxia leads to increased urea loss and disruptions in acid-base and ionoregulatory balance in dogfish sharks (*Squalus acanthias*). Physiol Biochem Zool 87: 623–639. 10.1086/677884. 25244375

